# Immediate effect of kinesiology taping on muscle strength, static balance and proprioception after eccentric muscle fatigue on ankle: a randomized cross-over trial

**DOI:** 10.1186/s12891-024-07365-6

**Published:** 2024-03-28

**Authors:** Yongjie Li, Yuan Xia, Dakuan Zhang, Shenyu Fu, Mengling Liu, Xinyong Pan, Hongju Liu

**Affiliations:** 1 Department of Rehabilitation Medicine, Beijing Jishuitan Hospital Guizhou Hospital, Guizhou Provincial Orthopedics Hospital, Guiyang, China; 2 Department of Rehabilitation Medicine, the affiliated hospital of Hubei Provincial government, Wuhan, China; 3 School of Health Sciences, Wuhan sports University, Wuhan, China

**Keywords:** Kinesiology tape, Ankle, Balance, Muscle strength, Muscle fatigue

## Abstract

**Background:**

Kinesiology Taping(KT) is commonly used as a physical therapy to prevent exercise-induced fatigue. This study aims to evaluate the immediate effects of KT on muscle strength, static balance, and proprioception after eccentric muscle fatigue on ankle.

**Methods:**

Twenty healthy male university students were recruited. The experimental protocol was structured into four sessions, each separated by a one-week washout period to prevent carryover effects. Participants were randomly allocated to one of four intervention conditions in each session, ensuring no participant received the same intervention twice. These conditions were: no taping(NT),sham taping(ST),athletic taping(AT),and kinesiology taping(KT).Taping was applied immediately following an eccentric muscle fatigue protocol targeting the ankle, and assessments were conducted in the order of proprioception, muscle strength and static balance. Isometric muscle strength and proprioception were evaluated using the Biodex isokinetic system. Static balance was measured using the TecnoBody balance platform.

**Results:**

KT had a significantly higher plantarflexion/dorsiflexion peak torque, dorsiflexion average peak torque, and plantarflexion/dorsiflexion average power at 60°/s compared with NT and ST in terms of isometric muscle strength (*p* < 0.05).Furthermore, the plantarflexion peak torque of KT was significantly greater than AT at 60°/s[*p* = 0.005,95% confidence interval(CI) = 3.39 to 18.20] and 180°/s[*p* = 0.006,95%CI(2.62,21.98)]. In terms of proprioception, KT showed a lower absolute error in 25° plantarflexion and 10° dorsiflexion compared to NT, ST and AT. For static balance with eyes-open and eyes-closed conditions, AT and KT had a lower total sway area than NT and ST (*p* < 0.05). Additionally, a significant difference in total sway length with eyes-open condition was observed between AT and KT[*p* < 0.001,95%CI(-431.81,-168.25)];total sway area and the center of pressure(COP) velocity in the mediolateral(ML) and anteroposterior(AP) directions with eyes-closed condition were significantly lower in AT compared to KT.

**Conclusion:**

This study suggests that KT is more effective than other taping conditions in improving muscle strength and proprioception after eccentric muscle fatigue on ankle. However, AT is more helpful in increasing static postural control ability after ankle muscle fatigue than KT.

**Trial registration:**

This study was registered with www.chictr.org.cn (registration number: ChiCTR2300068278) on 13/2/2023.

## Introduction

The ankle joint, situated at the distal part of the human lower extremity, is responsible for supporting body weight, absorbing impact, and maintaining balance. During physical activity, muscle fatigue can occur in the ankle joint as a result of repetitive muscle contractions or extended periods of high-intensity exercise [1]. Muscle fatigue is prevalent in competitive sports and is primarily characterized by diminished responsiveness, joint stiffness, decreased proprioception, and reduced postural control, which ultimately impairs athletic performance [2]. Furthermore, some studies [3, 4] have demonstrated that muscle fatigue diminishes neuromuscular control and increases ankle joint loading, thereby significantly elevating the risk of joint injury. Among the various factors contributing to muscle fatigue (e.g., sleep quality, immunity, type of muscle activity), the type of muscle activity stands out as a critical element. Research [5, 6]has indicated that distinct muscle contraction patterns can induce muscle fatigue, with eccentric contraction notably affecting skeletal muscle cells and being more prone to inducing muscle fatigue. Therefore, devising recovery strategies for muscle fatigue resulting from eccentric contraction has emerged as a key area of interest in sports medicine.

Various strategies exist for recuperating from muscle fatigue, among which physiotherapy stands out as a particularly popular option, attributing to its quick efficacy and the minimal side effects [7]. Kinesiology taping (KT) has emerged as a popular physiotherapy technique in musculoskeletal rehabilitation [8, 9]. KT primarily consists of elastic cotton fibers, approximately the same thickness as the skin’s epidermal layer, capable of stretching longitudinally up to 140% of their original length [10]. The fundamental mechanism of KT involves stretching the skin and soft tissue via the tape’s recoiling force, which stimulates the skin’s proprioceptors and increases sensory input [11]. Several studies [12–14]have reported the positive effects of KT on pain relief, muscle strength enhancement, and improved pain-free range of motion. Furthermore, some researchers [15–17] have found that KT can increase subcutaneous tissue space, thereby augmenting blood and lymphatic circulation and reducing swelling.

In recent years, a few studies [18–20] have investigated the effect of KT on the postural balance after ankle muscle fatigue, employing muscle fatigue protocols induced by concentric contraction and evaluating postural control ability. The studies by Choi and Farquharson [18, 19]suggested that KT has an immediate positive effect on static balance, while Li et al [20]. found that KT did not improve static balance parameters such as center of pressure (COP) sway range, COP velocity and sway area following ankle muscle fatigue. The potential reason for this discrepancy is the variation in KT application techniques (i.e., facilitatory KT and ankle balance KT) and degree of muscle fatigue. Moreover, Li et al. demonstrated that KT was insufficient to positively influence muscle strength and proprioception after ankle muscle fatigue [20]. In summary, previous studies employed muscle fatigue protocols induced by concentric contraction, leaving the effects of KT on muscle fatigue caused by eccentric contraction unclear. The primary outcome measures concentrated on postural control, leading to mixed results. Considering the complex influence of muscle fatigue on exercise performance, it becomes crucial to thoroughly assess the impact of KT on muscle strength, static balance, and proprioception deficits caused by ankle muscle fatigue. Therefore, the aim of our study was to evaluate the immediate effect of KT on muscle strength, static balance, and proprioception following eccentric muscle fatigue on the ankle. We hypothesized that KT would exert a beneficial immediate impact on muscle strength, static balance, and proprioception in individuals with healthy ankles experiencing eccentric muscle fatigue.

## Methods

### Sample size

The calculation of the estimated sample size was conducted using G*Power (version 3.1.9.7, Germany).This process was grounded in the outcome variable of plantarflexion peak torque at 60°/s, derived from a prior study [20].We opted for the F test within ANOVA (repeated measures) as our analytical approach. The sample size estimation was based on an effect size of 0.25, the α level of 0.05, a statistical power of 0.8, and accounted for a dropout rate of 0.20. Consequently, it was determined that the minimum sample size required is at least 20 subjects.

### Subjects

Healthy volunteers were recruited from a nearby university and community from public announcements, as well as from an existing list of interns at our hospital. A total of twenty male healthy university students with running habits (10–20 km per week) were ultimately enrolled in the study. Inclusion criteria were as follows: (1) age from 18 to 25 years;(2)qualified heart rate(60-100beats per minute), blood pressure(90mmHg < systolic pressure < 140mmHg, 60mmHg < diastolic pressure<90mmHg), and body mass index(BMI,18.50-23.99 kg/m^2^) [21]; (2)passing the Physical Activity Readiness Questionnaire (PAR-Q) test [22]; (3)no musculoskeletal disorders within the last 3 months and intact skin on the ankle ;(4)no neurological or circulatory disease; (5)no additional exercise or related treatment during the experimental cycle; (6) no exercise-induced fatigue generated before the experiment; (7)right-sided dominant leg. Exclusion criteria included:(1) acute soft tissue injury in the lower extremity within the last month; (2) allergy to KT and athletic taping (AT); (3) history of surgical procedures; (4) functional impairment of vision and vestibular sensation. Table 1 presents the demographic characteristics of subjects.

We confirmed that all methods were performed in accordance with the relevant guidelines and regulations of the Helsinki declaration. This study was approved by the ethics committee of Beijing Jishuitan Hospital Guizhou Hospital(approval number: LW20221101), and was registered with www.chictr.org.cn (registration number: ChiCTR2300068278,13/2/2023). In addition, informed consent was obtained from all the subjects.

**Table 1:** Demographic characteristics of subjects (Mean values ± SD).

Sample size	Age (years)	Height (cm)	Weight (kg)	BMI (kg/m^2^)
20	21.70 ± 0.86	169.45 ± 6.76	62.85 ± 7.68	21.85 ± 2.28

### Study design

**Fig. 1:**
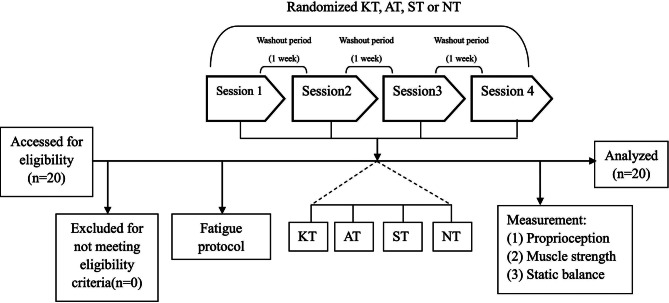
The flowchart diagram of this study

This study employs a four-intervention, randomized, crossover design, comprising no taping (NT), sham taping (ST), athletic taping (AT), and kinesiology taping (KT). The research protocol involved four sessions, each separated by a one-week washout period to ensure no carryover effects. A professional statistician, who was not involved with the study, used a random number generator (https://www.randomizer.org/) to create a sequence of random numbers, which were then sealed in a non-transparent envelope. An independent researcher utilized these numbers to randomly assign participants to one of the intervention types for each session, avoiding any repetitions. A preliminary familiarization session was held three days prior to the experiment to introduce the participants to the procedures. The intervention protocols, consisting of the four taping conditions, were applied immediately after subjects completed an eccentric muscle fatigue protocol targeting the ankle. The assessments were conducted sequentially, starting with proprioception, followed by muscle strength, and ending with static balance. After completing the proprioception test, participants had a 30-second rest before proceeding to the muscle strength test, and similarly, a 30-second rest before the static balance test. Assessment of outcomes was carried out by a physiotherapist blinded to the intervention types, and an independent statistician was responsible for the data analysis. The methodology and flow of the study are illustrated in Fig. 1.

### Fatigue protocol

The fatigue protocol was conducted on a Biodex isokinetic system (Biodex Medical System,USA) following two previous studies [23, 24]. The eccentric/eccentric training mode was selected, and consecutive maximal plantarflexion/dorsiflexion contractions were performed at an angular velocity of 120°/s. The average of the first three plantarflexion/dorsiflexion peak torque was defined as the maximum initial peak torque (IPT). Fatigue was considered to have occurred when torque output in both directions was lower than 50% IPT for three consecutive times.

### Taping methods

**Fig. 2:**
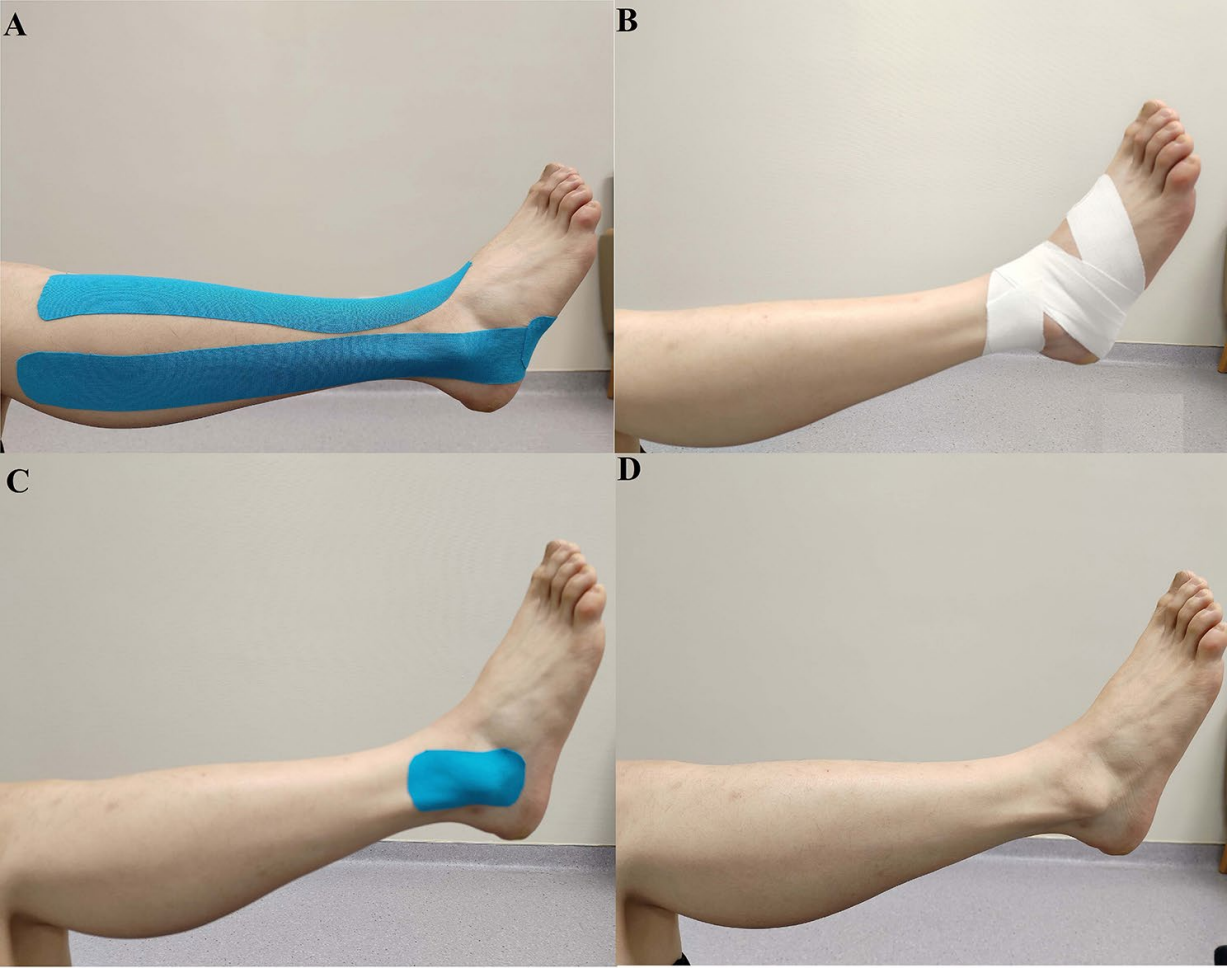
Application of four tapingtreatments.(A) kinesiology taping, KT; (B) athletic taping, AT;(C)sham taping, ST;(D)no taping, NT.

Intervention types were no taping (NT), sham taping (ST), athletic taping (AT), kinesiology taping (KT)(Fig. 2).

#### KT

For the KT intervention, we utilized kinesiology tape (Kindmax, 50 mm x 5 m, China) applying the facilitation technique (50% tension) as outlined by Jackson et al. [25]. This method comprised four distinct steps: (1) The initial strip was placed at the origin of the anterior tibial muscle, extended downwards following the muscle’s pathway, and terminated near the first cuneiform and first metatarsal bones; (2) The second strip began at the inner posterior part of the tibia and fibula, descended along the muscle’s course, and ended near the medial malleolus; (3) The third phase involved starting the third strip around the fibular head, leading it to the base of the first metatarsal via the lateral malleolus; (4) The final step involved applying the fourth strip, starting just anterior to the lateral malleolus, extending across the transverse arch of the plantar, and finishing anterior to the medial malleolus.

#### AT

For the AT intervention, athletic tape (AOPI, 50 mm x 9.1 m, China) was used, adhering to the technique described by Choi and Yin et al [19, 26]. This approach consisted of four steps: (1) With the ankle in slight dorsiflexion to enhance posterior talar glide [27], the strip was applied from the middle of the dorsal talus towards the calcaneus bone; (2) While maintaining the ankle in an inversion position, the second strip was positioned, starting 5 cm above the medial malleolus, extending over the lateral malleolus, and then towards the lateral plantar area; (3) With the ankle in eversion, the third strip commenced 5 cm above the lateral malleolus, crossed over the medial malleolus to the medial plantar area; (4) The procedure was concluded by repeating the first step.

#### ST

Tension-free KT was applied to the medial and lateral malleolus without involving the muscle’s origin and insertion.

### Outcome measures

#### Muscle strength

Muscle strength was assessed using the Biodex isokinetic system. During the test, the subject’s foot was securely fastened to the dynamometer footplate, and the ankle joint was positioned neutrally. The ankle joint range of motion in the dorsiflexion/plantarflexion direction was set from − 20° to 50°, with angular velocities of 60°/s and 180°/s. Plantarflexion/dorsiflexion contractions were alternated, consisting of five repetitive movements and a one-minute interval between different angular velocity tests. Laboratory test indicators included plantarflexion/dorsiflexion peak torque(Nm), average peak torque(Nm), and average power (W) at 60°/s and 180°/s.

#### Proprioception

Proprioception was evaluated through joint position perception using the Biodex isokinetic system. The passive position sense mode was selected, with target positions set at 10° dorsiflexion and 25° plantarflexion [28]. The ankle joint was positioned at 0° as the baseline for each test, with each target position being assessed three times. The Biodex isokinetic system moved the subject’s ankle from this baseline to the target position, holding it there for 10 s to allow the subject to sense the joint’s position. To eliminate visual cues, subjects were blindfolded throughout the procedure. After each sensing period, the foot was returned to the starting position, and the system then moved the ankle toward the target position at a rate of 1°/s [29]. When the subject believed they had reached the target position, they immediately pressed the “stop” button.

The absolute error(AE) between the actual stopping angle and the target angle was employed to reflect joint position perception levels. The average of the three AE values was used as the final result, with larger values indicating poorer proprioception.

#### Static balance

**Fig. 3:**
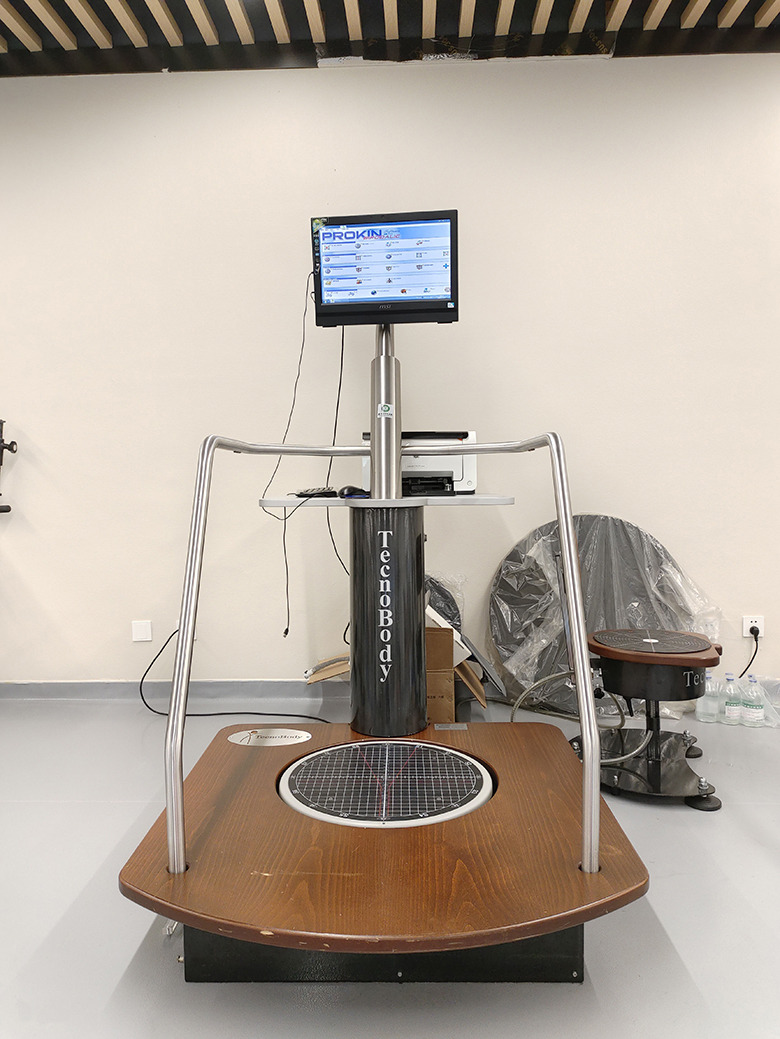
TecnoBody balance platform

Single-leg static balance assessments, both with eyes open and closed, were conducted using the TecnoBody balance platform (TecnoBody, PK252, Italy) as illustrated in Fig. 3. Participants stood on their right foot at the center of the pressure platform, left knee bent at 90°, and arms hanging naturally. Measurements were taken while participants tried to maintain their stance as long as possible under both eyes-open and eyes-closed conditions. Each condition involved two trials, with each lasting 30 s and a 15-second rest period between trials. Laboratory indicators included mediolateral (ML) COP sway range (mm), anteroposterior (AP) COP sway range (mm), ML COP velocity(mm/s), AP COP velocity(mm/s), total sway area (mm^2^), and total sway length(mm), with lower values indicating better static balance. These parameters, known for their reliability, comprehensively represent static balance ability and show marked differences between eyes open and closed conditions [30], justifying their selection for this study.

### Statistical analysis

Statistical analyses were conducted using SPSS software version 21.0 (IBM, USA). The dataset comprised continuous variables, which were presented as mean ± standard deviation (SD). The Shapiro-Wilk test was utilized to verify the normal distribution of the data. For comparing differences between groups, one-way repeated measures ANOVA was applied. In cases where the test for homogeneity of variance was met, the Bonferroni correction was used for multiple post hoc comparisons. Conversely, if the variance was found to be non-homogeneous, the Games-Howell method was applied for multiple post hoc analyses. A significance level of *P* < 0.05 was established for all statistical tests [26, 31].

## Results

### Muscle strength

**Fig. 4:**
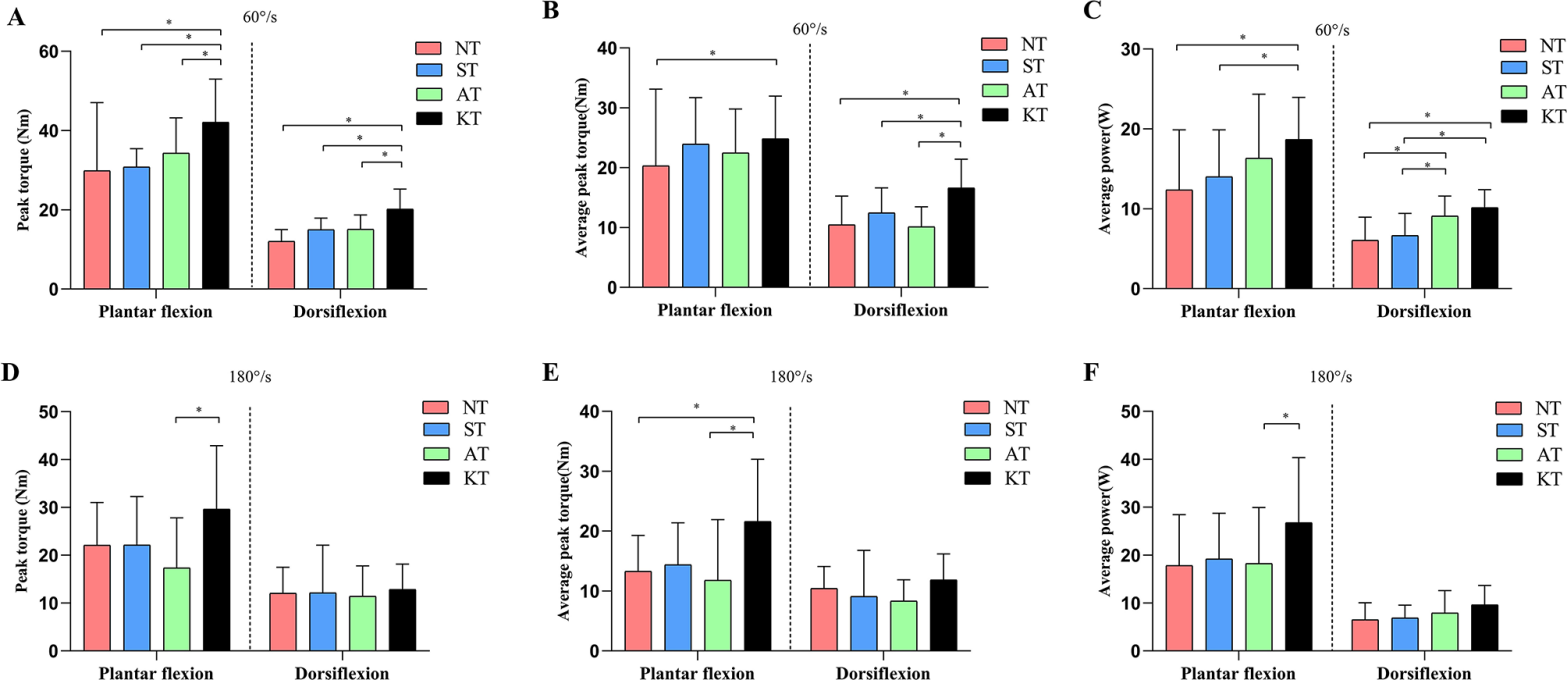
Comparison of isokinetic muscle strength variables at 60°/s and 180°/s among four taping conditions.(A) peak torque at 60°/s ;(B)average peak torque at 60°/s; (C)average power at 60°/s; (D) peak torque at 180°/s ;(E)average peak torque at 180°/s; (F)average power at 180°/s.*p < 0.05,the difference was statistically significant

Figure 4 displays the muscle strength results. At 60°/s, a significant difference was observed among the four taping interventions in all outcome measures. Post-hoc analysis indicated that the plantarflexion/dorsiflexion peak torque, dorsiflexion average peak torque, and plantarflexion/dorsiflexion average power were significantly higher in KT compared to NT and ST(*p* < 0.05).The plantarflexion/dorsiflexion peak torque[**plantarflexion**:*p* = 0.005, 95%CI(3.39,18.20); **dorsiflexion**: *p* < 0.001, 95%CI( 2.65,7.53)] and dorsiflexion average peak torque [*p* < 0.001, 95%CI(3.50,9.48)]of KT was significantly higher than AT.

At 180°/s, a significant difference was observed among the four taping interventions in plantarflexion with peak torque(F = 4.090,*p* = 0.010,η^2^ = 0.146), average peak torque(F = 4.759,*p* = 0.004,η^2^ = 0.171) and average power(F = 4.502,*p* = 0.006,η^2^=.

0.158). Post-hoc analysis indicated that the plantarflexion average peak torque of KT was significantly higher than NT [*p* = 0.034, 95% CI(0.39, 16.29)]. In addition, the plantarflexion peak torque[*p* = 0.006, 95% CI(2.62, 21.98)], plantarflexion average peak torque[*p* = 0.006, 95% CI(2.12, 17.53)] and plantarflexion average power [*p* = 0.003, 95% CI(3.34, 23.73)]of AT were significantly lower than KT.

### Proprioception

**Fig. 5:**
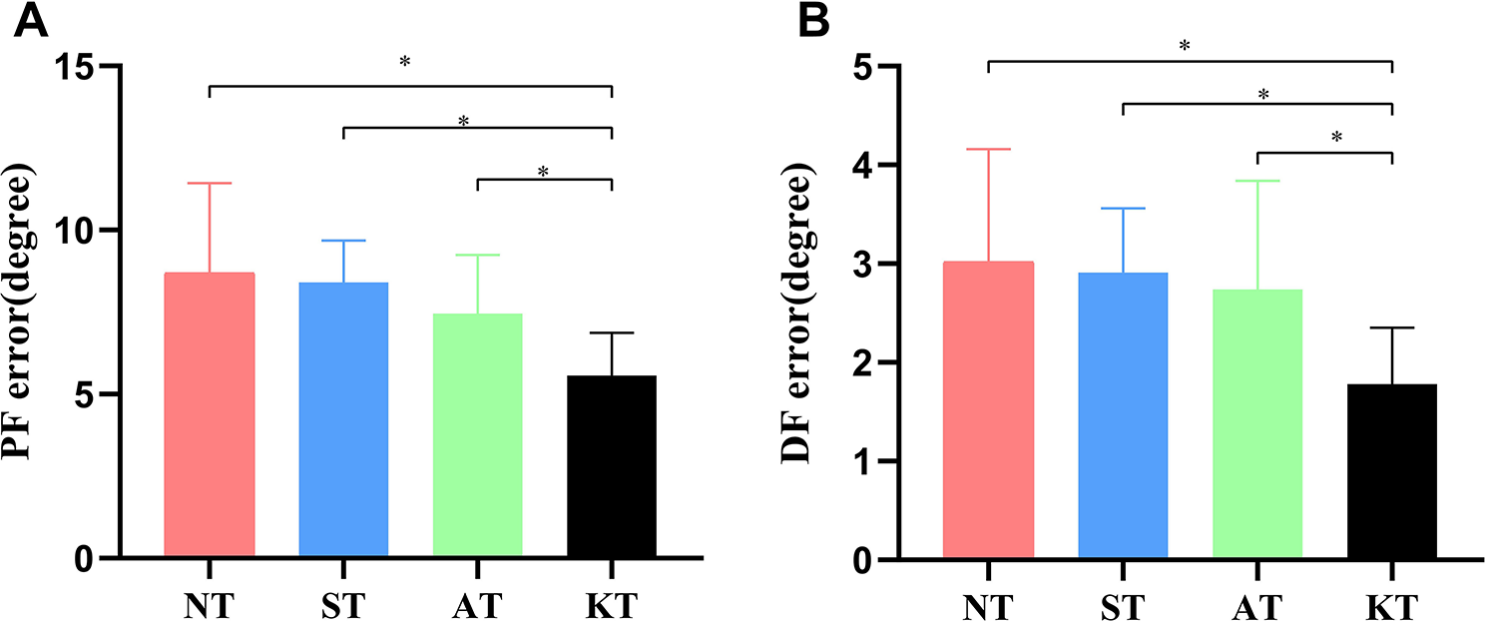
Comparison of absolute error values in ankle joint position among four taping conditions. (A) 25◦ plantarflexion(PF) (B) 10◦ dorsiflexion (DF),* *p* < 0.05,the difference was statistically significant

Figure 5 shows the results of proprioception(absolute error values). A significant difference was found among the four taping interventions in 25° plantarflexion (F = 11.053,*p* < 0.001,η^2^ = 0.317) and 10° dorsiflexion (F = 6.93,*p* < 0.001,η^2^. = 0.224).Post-hoc analysis indicated that Kt showed a lower absolute error (AE) in 25° plantarflexion and 10° dorsiflexion compared to NT[25° plantarflexion:*p* < 0.001, 95% CI( -4.33,-1.91);10° dorsiflexion:*p* < 0.001, 95% CI( -1.72,-0.56)], ST[25° plantarflexion:*p* < 0.001, 95% CI( -4.01,-1.65);10° dorsiflexion: *p* < 0.001, 95% CI( -1.69,-0.55)] and AT[25° plantarflexion:*p* = 0.003, 95% CI( -3.10,-0.68);10° dorsiflexion: *p* = 0.002, 95% CI( -1.54,-0.37)].

### Static balance

Table 2 presents the static balance results with eyes open. No statistically significant difference was observed among the four taping interventions in ML COP sway range(F = 0.327,*p* = 0.806, η^2^ = 0.013) and ML COP velocity(F = 1.878,*p* = 0.141,η^2^ = 0.07). Post-hoc analysis revealed that the total sway area[AT vs. NT: *p* < 0.001, 95% CI (-1560.37, -848.11);KT vs. NT: *p* < 0.001, 95% CI (-1208.05, -504.95)] and total sway length[AT vs. NT:*p* < 0.001,95% CI (-632.29, -361.28);KT vs. NT: *p* = 0.005, 95% CI(-330.51, -63.00)] of AT and KT were significantly lower than NT. Both KT and AT demonstrated lower AP COP velocity [AT vs. ST: *p* = 0.010, 95% CI (-21.04, -3.01);KT vs. ST:*p* = 0.043, 95% CI (-17.66, -0.12)] and total sway area compared to ST. Additionally, a significant difference in total sway length was observed between AT and KT [*p* < 0.001, 95% CI (-431.81, -168.25)].

Table 3 presents the static balance results with eyes closed. A significant difference was observed among the four taping interventions in all outcome measures(F = 5.632 to 39.837, *p* < 0.05, η²=0.190 to 0.631). Post-hoc analysis demonstrated that AP COP velocity, total sway area, and total sway length of KT and AT were significantly lower than NT and ST (*p* < 0.05). Furthermore, the ML COP velocity [*p* < 0.001, 95% CI (-30.91, -13.34)], AP COP velocity[*p* < 0.001, 95% CI (-34.82, -18.87)], and total sway length [*p* < 0.001, 95% CI (-828.75, -18.87)] of AT were significantly lower than KT.

**Table 2:** Comparison of static balance with eyes open among four taping conditions (Mean values ± SD).

Intervention	ML COP sway range (mm)	AP COP sway range (mm)	ML COP velocity(mm/s)	AP COP velocity (mm/s)	Total sway area (mm^2^)	Total sway length(mm)
NT	5.84 ± 4.72	7.47 ± 5.40	34.61 ± 21.73	34.44 ± 26.79	2105.29 ± 772.88	1383.18 ± 180.97
ST	5.00 ± 2.29	4.75 ± 2.65	32.40 ± 5.85	36.30 ± 5.06	1925.95 ± 319.80	1224.85 ± 198.61
AT	5.72 ± 2.27	5.00 ± 2.11	26.22 ± 5.23	24.27 ± 6.34^†^	901.06 ± 427.89*^†^	886.39 ± 246.24*^†^
KT	5.26 ± 1.97	4.16 ± 1.86*	28.47 ± 6.80	27.52 ± 4.59^†^	1248.80 ± 521.76*^†^	1169.03 ± 264.58*^‡^
*F* values	0.327	3.714	1.878	3.121	20.771	18.880
*P* values	0.806	0.015	0.141	0.031	0.001	0.001
η^2^	0.013	0.134	0.07	0.116	0.470	0.447

*Note*: COP = center of pressure; ML = mediolateral;AP = anteroposterior.

*Indicates a difference compared with NT (*p*<0.05);

^†^Indicates a difference compared with ST (*p*<0.05);

^‡^ Indicates a difference compared with AT (*p*<0.05).

**Table 3:** Comparison of static balance with eyes closed among four taping conditions (Mean values ± SD).

Intervention	ML COP sway range (mm)	AP COP sway range (mm)	ML COP velocity (mm/s)	AP COP velocity (mm/s)	Total sway area (mm^2^)	Total sway length(mm)
NT	13.37 ± 12.11	16.61 ± 10.20	63.27 ± 12.65	67.88 ± 12.65	4593.65 ± 1821.49	3519.76 ± 1188.36
ST	10.20 ± 2.91	11.40 ± 4.19	65.50 ± 14.46	66.30 ± 13.91	4144.60 ± 880.32	3266.95 ± 571.27
AT	4.83 ± 2.92*^†^	6.56 ± 6.44*	35.61 ± 11.27*^†^	28.89 ± 12.25*^†^	2285.67 ± 598.86 *^†^	2017.78 ± 461.62*^†^
KT	7.84 ± 2.93^‡^	10.47 ± 6.63	57.73 ± 9.51*^‡^	55.73 ± 9.30*^†‡^	2855.53 ± 768.61 *^†‡^	2373.52 ± 469.83*^†^
*F* values	6.090	5.632	24.182	39.837	14.208	17.717
*P* values	0.001	0.002	0.001	<0.001	<0.001	<0.001
η^2^	0.204	0.190	0.509	0.631	0.378	0.431

*Note*: COP = center of pressure; ML = mediolateral;AP = anteroposterior.

*Indicates a difference compared with NT (*p*<0.05);

^†^Indicates a difference compared with ST (*p*<0.05);

^‡^ Indicates a difference compared with AT (*p*<0.05).

## Discussion

This study aimed to investigate the immediate effect of KT on muscle strength, static balance, and proprioception following eccentric muscle fatigue on ankle. The findings revealed that, in comparison to AT, KT demonstrated greater efficacy in enhancing muscle strength and proprioception after eccentric muscle fatigue on ankle. However, AT outperformed KT in improving static balance.

### Effect of KT on muscle strength

Muscle strength plays a crucial role in joint flexibility and stability and is an essential component of fatigue recovery programs [32]. In this study, isometric muscle strength tests were conducted at 60°/s and 180°/s angular velocities. The results indicated that the majority of isometric muscle strength indicators for plantarflexion and dorsiflexion in the KT group improved significantly at a 60°/s angular velocity compared to other taping techniques. However, there was a trend towards a difference at a 180°/s angular velocity(*p* > 0.05). These findings suggest that KT may effectively promote muscle strength recovery following eccentric muscle fatigue, which could be associated with the recoiling force direction of the tape. In this study, the recoiling force direction of KT was aligned with the direction of muscle centripetal contraction, which was more conducive to activating the γ-motor neurons in soft tissues. Consequently, this led to changes in muscle fiber tension that promoted muscle contraction, ultimately resulting in enhanced muscle strength [33]. A study [34] highlighted that KT consistently activates skin mechanoreceptors and improves neuromuscular recruitment, thereby exerting a positive effect on muscle strength. Moreover, eccentric contraction generates increased levels of substances such as lactate and creatine kinase, which contribute to muscle fatigue. KT can promote lymphatic circulation and blood flow by expanding the subcutaneous space, which assists in the elimination of excess lactic acid and creatine [35].

Notably,the major findings regarding muscle strength in this study are in contrast to previous studies. Gómez-Soriano and Lumbroso [36, 37] reported that KT did not enhance the muscle strength of plantarflexion and dorsiflexion. Upon comparison, it was observed that their studies employed the insertion-to-origin taping method to inhibit muscle function and involved healthy subjects rather than those in a fatigued state. In contrast, our study utilized facilitatory KT technique, and all subjects underwent a fatigue protocol. As such, the differences in KT methods and the subjects’ states might be the sources of heterogeneity in the study results.

### Effect of KT on proprioception

Proprioception, also known as deep sensation, encompasses kinesthetic, positional, and vibratory sensations, and plays a crucial role in controlling human movement [38]. In current research, proprioception quantification is often reflected by position perception, which represents the absolute error value of joint angles. Previous studies [39]have demonstrated that a reduction in proprioception sensitivity significantly increases the risk of ankle injury, leading to the proposal of strengthening proprioception. The results of this study indicated that KT outperformed other taping methods in reducing the AE values of both ankle plantarflexion and dorsiflexion, suggesting that KT can enhance proprioceptive ability following eccentric muscle fatigue on the ankle. The significant improvement in proprioception might be attributed to the mechanoreceptor stimulation provided by KT, which counteracts the sensory input deficiency caused by fatigue [40]. Riemann [41] posited that applying pressure or stretching the skin could stimulate skin mechanoreceptors, which play a vital role in detecting joint motion and position. Our findings in this regard are supported by Brogden and Yu [42, 43], who reported that KT can enhance sensory input to the ankle joint, resulting in improved proprioception. However, two studies [20, 44]presented results that were inconsistent with our findings, suggesting that KT cannot be used to improve ankle joint proprioception. Upon comparison, it was observed that Li et al. [20]assessed proprioception using position perception (5° of plantarflexion and 5° of dorsiflexion), but the target position angle set in their study differed significantly from ours. Bailey et al. [44]evaluated proprioception through balance and fine movement control tasks rather than position perception. In summary, different proprioception testing methods may be an important factor in these inconsistent results.

The study found AT did not enhance proprioception post-fatigue, potentially due to AT’s rigid support limiting joint movement. Proprioception was measured using the Biodex isokinetic system, which requires extensive joint movement, conflicting with AT’s non-extensible nature. AT’s function is to provide fixed support to the joint without extensibility, limiting the range and sensitivity of joint movement and potentially having a negative impact on proprioception. Given that only position sense was used to evaluate proprioception in this study, future research should further examine the effects of different taping conditions on proprioception following fatigue.

### Effect of KT on static balance

Postural control is dependent on body balance, a fundamental requirement for movement and activities of daily living [1, 45]. Previous research [46, 47] has indicated that the negative consequence of decreased balance ability due to fatigue is an increased risk of ankle injury, which accounts for nearly 26% of sports injury types. This study examined the impact of KT on static balance after eccentric muscle fatigue on the ankle, revealing a positive effect of KT in enhancing static balance in both eyes-open and eyes-closed conditions, consistent with the findings of Park and Lin et al. [48, 49]. The underlying physiological mechanism could involve the facilitatory KT improving the neuromuscular capacity of the muscles surrounding the ankle, thereby promoting muscle fiber recruitment and increasing joint stability to achieve body balance [50]. KT’s improvement of static balance might also be attributed to increased joint stiffness. Joint stiffness is calculated based on the ratio of the change in torque to the change in joint angle [51], and our study has demonstrated that KT improved plantarflexion/dorsiflexion peak torque. In terms of joint angle, KT provided external support to the muscle tissue around the ankle joint, limiting the target muscle’s ability to radially shift during contraction [52]. This additional resistance to radial expansion likely increased joint stability during static stance and reduced the ankle joint’s angle variation [52, 53]. In summary, the increase in ankle joint torque, accompanied by a decrease in joint angle change, ultimately contributed to increased joint stiffness. Furthermore, it is noteworthy that large SD values were observed in the NT condition but were smaller for the ST condition, consistent with the study reported by Li et al. [20]. Our finding could be associated with the placebo effect. Mak et al. and de-la-Torre-Domingo et al. [54, 55]suggested that Sham KT had a placebo effect, contributing to a positive outcome by increasing subject confidence. Unlike the NT in the present study, Sham KT was used in the ST, and the taping method, as a psychological cue, might have led to increased expectations and confidence in subjects, thereby enhancing their engagement during the test. The placebo effect, to some extent, helped subjects perform true motor performance. Individual differences (the degree of dispersion of test data) were reduced, so SD values were lowered.

This study also discovered that static balance outcome measures were significantly higher in the eyes-closed condition than in the eyes-open condition, suggesting that vision occlusion could have a considerable impact on balance. Static balance is regulated by visual, vestibular, and proprioceptive feedback [56]. In the eyes-closed condition, the body relies mainly on vestibular and proprioception without visual involvement, which better reflects the body’s ability to control balance [57, 58]. Jeon et al. [59]compared the differences in balance function in older adults standing on one leg before and after the removal of visual input, demonstrating that the absence of visual input increased postural instability, leading to a significant decline in balance.This also indicated that visual information is crucial for balance function. Interestingly, AT exhibited a better effect than KT. The primary reliance on isometric muscle contraction in maintaining static balance does not necessitate the generation of additional joint activity. Although KT possesses excellent extensibility, this property could limit its effect on static balance [60]. AT, being non-elastic, offers a superior stabilizing and supportive effect on the joint, similar to an ankle brace, which significantly strengthens joint stability and limits the range of motion to increase joint stiffness, ultimately promoting static balance [61, 62]. In summary, this study suggests that AT is more effective than KT in improving static balance.

### Limitations

The study has limitations, including: (1) Only the immediate effects of KT were investigated, leaving its long-term effects unclear. (2) The effects were observed only in a healthy population, suggesting a need for research across different populations. (3) Given that our study exclusively involved male subjects, it is essential to replicate this trial with female participants to obtain a more comprehensive understanding of the intervention’s effectiveness across genders.

## Conclusion

The study showed KT significantly enhanced muscle strength and proprioception after eccentric muscle fatigue on the ankle more than other taping methods. Furthermore, KT surpassed AT in improving static postural control post-ankle muscle fatigue. These insights can guide healthy individuals in choosing the suitable taping technique to quickly recover muscle strength, proprioception, and static balance affected by eccentric contraction-induced ankle muscle fatigue, thus lowering the risk of ankle injuries.

## Data Availability

The datasets used and/or analysed during the current study are available from the corresponding author on reasonable request.

## Abbreviations

KT: Kinesiology Taping
NT: No taping
ST: Sham taping
AT: Athletic taping
CI: Confidence interval
AE: Absolute error
COP: Center of pressure
AP: Anteroposterior
ML: Mediolateral
PAR-Q: Physical Activity Readiness Questionnaire
IPT: Initial peak torque
